# Phytate degradation and pre-cecal phosphorus digestibility of raw vs. crimped-ensiled faba beans (*Vicia faba* L.) fed to broiler chickens

**DOI:** 10.1016/j.psj.2025.106157

**Published:** 2025-11-24

**Authors:** Letícia Soares, Heidi Högel, Liisa Keto, Marcia Franco, Marketta Rinne, Markus Rodehutscord, Gabriel da Silva Viana

**Affiliations:** a Production Systems, Natural Resources Institute Finland, Jokioinen 31600, Finland; b Institute of Animal Science, University of Hohenheim, Stuttgart 70599, Germany

**Keywords:** Phytate degradation, Phosphorus digestibility, Fermentation, Leguminous crop

## Abstract

The current study was conducted to determine the effects of crimping and ensiling process on the pre-cecal disappearance of myo-inositol hexakisphosphate (InsP_6_) and the pre-cecal digestibility (pcd) of total phosphorus (P) of faba bean seeds (cv. Vire) fed to broiler chickens. On d 21 post-hatch, 360 male Ross308® broiler chicks were randomly assigned to one of six treatments, each with six replicates of 10 chicks, for a 7-day assay. The experimental treatments consisted of six diets (mash) containing either raw [5.5 g P/kg dry matter (DM); 9.37 g InsP_6_/kg DM] or crimped-ensiled (5.7 g P/kg DM; 8.45 g InsP_6_/kg DM) faba bean seeds (dried and milled prior to feeding) at inclusion levels of 260, 403, and 546 g/kg. Analyzed P content ranged from 2.37 to 4.09 g/kg DM in raw faba bean feeds and 2.64 to 4.41 g/kg DM in crimped-ensiled faba bean feeds. Dietary InsP_6_ content increased from 2.51 to 5.02 and 5.08 g/kg DM in raw and crimped-ensiled faba bean feeds, respectively. On d 28 post-hatch, all birds were sacrificed, and digesta samples were collected from the distal ileum. Increasing faba bean seed inclusion in feeds linearly decreased the pre-cecal disappearance of InsP_6_ in broiler chickens (*p* < 0.01) and values were lower in birds fed crimped-ensiled faba bean seeds (36.8 vs. 46.0 %). Irrespective of the processing method, faba bean inclusion linearly decreased the apparent pcd of P of feeds (*p* < 0.01), but birds fed crimped-ensiled beans showed higher pcd of P (*p* < 0.01) compared to those fed raw seeds (72.1 vs. 61.3 %). The pcd coefficients of P for raw and dried crimped-ensiled faba bean seeds (cv. Vire) were 42 % and 49 %, respectively. Increasing faba bean inclusion and dietary P concentration improved performance responses (*p* < 0.01), and broilers fed crimped-ensiled faba beans exhibited better performance compared to birds fed raw seeds (*p* < 0.01). The crimping-ensiling process increased the pre-cecal disappearance of InsP_6_ and the pcd of P in faba bean seeds when fed to broiler chickens.

## Introduction

Faba bean (*Vicia faba* L.) is a leguminous protein crop cultivated in Northern Europe, where the harsh weather conditions restrict the cultivation of crops traditionally used in poultry feeds such as soya bean (*Glycine max*) (Stoddard, 2017; Rahate et al., 2021; Staniak et al., 2023). Despite its satisfactory content of crude protein and starch, faba beans have been used at low levels in poultry feeds due to thermo-resistant antinutritional factors (ANF) like vicine and convicine (V + C), which adversely affect bird health and welfare (Muduuli et al., 1982; Lessire et al., 2017). A new faba bean cultivar (cv. Vire) with low (V + C) concentrations was recently developed in Finland. Although the reduced V + C levels may increase the suitability of faba bean as a protein source in poultry diets, the digestible nutrient profile of cv. Vire remains unknown.

Similarly, as for other grain legumes, the majority of total phosphorus (P) in faba beans is stored as phytic acid [myo-inositol 1,2,3,4,5,6-hexakis(dihydrogen phosphate); InsP₆], and its salt, phytate, which is mainly located in globoids inside protein storage vacuoles (Reddy et al., 1982). In faba bean, InsP_6_-P might reach up to 5.72 g/kg dry matter (DM) (Hejdysz et al., 2022; Siegert et al., 2022; Perz et al., 2023). Although birds can partially dephosphorylate phytate molecule (Birge and Avioli, 1981; Maenz and Classen, 1998), the hydrolysis of phytate P in plant-based feeds is largely dependent on supplemental exogenous phytases. The digestive dynamics of phytate along the poultry gastrointestinal tract (GIT) and its antinutritional properties were recently reviewed by Rodehutscord et al. (2022). As detailed by the aforementioned authors, the activity of endogenous phytases in broiler GIT may be constrained by multiple factors, including the content of P and calcium (Ca) in feeds as the inclusion of feed inorganic phosphates and limestone. Evidence has suggested that in pigs the size of InsP isomer is correlated with its solubility in the anterior small intestinal lumen, whereby lower InsP forms exhibit higher solubility in the small intestine (Schlemmer et al., 2001). The lower InsP have been also shown to have attenuated antinutritive effects due to a reduced capacity of chelating positively charged nutrients in the GIT (Persson et al., 1998).

Phytate has been shown to be partially hydrolyzed through various processing methods (Alonso et al., 2000; Hejdysz et al., 2022). Previous findings indicate that soaking and fermentation degrade, to some extent, phytate in faba beans and barley (Perttilä et al., 2001; Luo et al., 2009), which may explain the improvements in P digestibility observed in fermented amylaceous grains fed to pigs and poultry (Pieper et al., 2011; Poulsen et al., 2012; Koo et al., 2018). Fermentation has been proposed as an effective approach to enhance the nutritional value of feed ingredients and reduce the activity of other antinutritional factors (ANFs) (Rinne et al., 2020; Rahate et al., 2021). Crimping and ensiling cereal grains is a practical and cost-effective method of seed preservation under humid conditions, offering a more economical and environmentally friendly alternative compared to seed drying (Jokiniemi et al., 2014). The preservation process involves crushing the moist seeds with a roller mill and compacting them anaerobically into a silo, allowing lactic acid fermentation to occur while preventing the growth of spoilage microbes. Although crimped-ensiled cereal grains and grain legume seeds have been extensively utilized in ruminant nutrition, their potential has not been fully explored in poultry nutrition. Fermentation can reduce cellulose content and increase soluble protein in plant-origin ingredients (Teng et al., 2017; Yeh et al., 2018; Zhang et al., 2022), potentially increasing the utilization of other nutrients in feeds. To the best of our knowledge, no previous studies have assessed whether, and to what extent, the crimping and ensiling process could improve the nutritional value of faba bean seeds in poultry nutrition. We hypothesized that crimping and ensiling would improve the digestibility of P in broilers. The aims of the current study were to 1) determine whether crimping and ensiling process can partially dephosphorylate phytate in faba beans and 2) evaluate the extent to which it affects the pre-cecal digestibility (pcd) of P in faba bean seeds fed to broiler chickens.

## Material and methods

### Faba bean seeds and processing methods

The faba bean seeds cv. Vire used in the current study were obtained from Boreal Plant Breeding Ltd. (Jokioinen, Finland). For raw seeds, the dry seeds were ground using a 2.0-mm screen sieve prior to inclusion in experimental diets. Seeds designated for processing were rehydrated with tap water to simulate the moisture content of freshly harvested beans (around 350 g moisture per kg) by adding 225 g water per kg of seeds one day prior to crimping. The moistened seeds were manually mixed 3 times on the previous day and once on the day of crimping to ensure that all water was absorbed, and the moisture was equally distributed within the batch. The moistening procedure was conducted in a hall at approximately 15°C temperature. The rehydrated seeds were then crimped (slightly crushed) using a pilot scale roller mill (Nipere Ltd., Teuva, Finland). Subsequently, a commercial heterofermentative lactic acid bacteria inoculant (Kofasil® Duo, Addcon GmbH, Bitterfeld-Wolfen, Germany) was manually applied and thoroughly mixed with the beans at the manufacturer’s recommended dosage [*Lactiplantibacillus plantarum* (DSM 3676, 3677) and *Lentilactobacillus buchneri* (DSM 13573) at 2.0 × 10^5^ cfu/g fresh beans]. Finally, the beans were carefully compacted into 60-liter plastic barrels, which were sealed airtight. The barrels were stored at room temperature (∼20°C) and opened after a fermentation period of approximately 2.5 months. The initial pH was 7.06 and at the time of opening, the pH of the feed was 4.64. For experimental diet manufacturing, the fermented beans were dried at 40°C in a forced air oven for approximately 48 hours until fully dry. Prior to the feeding trials, the dried crimped-ensiled faba bean seeds were ground using a 2.0-mm screen. The composition of the raw and crimped-ensiled seeds is detailed in Table 1.

**Table 1:** Analyzed nutrient and antinutritive factor concentrations of raw and crimped-ensiled faba bean seeds cv. Vire.

	Raw	Crimped-ensiled
Dry matter, g/kg	880	923
Gross energy, kcal/kg DM	4479	4505
Crude protein, g/kg DM	288	293
Crude fiber, g/kg DM	97.8	104.1
Neutral detergent fiber, g/kg DM	173.8	133.0
Acid detergent fiber, g/kg DM	108.1	103.2
Total indispensable amino acids, g/kg DM		
Arginine	26.9	20.5
Histidine	7.2	7.6
Isoleucine	10.9	11.6
Leucine	21.2	22.0
Lysine	20.7	20.7
Methionine	2.1	2.1
Phenylalanine	12.0	12.5
Threonine	10.3	10.6
Tryptophan	2.6	2.7
Valine	13.3	13.7
Ash, g/kg DM	38.4	38.8
Calcium, g/kg DM	1.5	1.5
Phosphorus, g/kg DM	5.5	5.7
InsP_6_-phosphorus, g/kg DM	2.64	2.38
InsP_6_, g/kg DM	9.37	8.45
Ins(1,2,4,5,6)P_5_, g/kg DM	0.93	0.65
Ins(1,2,3,4,5)P_5_, g/kg DM	0.25	0.29
Ins(1,3,4,5,6)P_5_, g/kg DM	LOD^1^	0.29
Ins(1,2,3,4,6)P_5_, g/kg DM	LOQ^2^	LOD
Ins(1,2,5,6)P_4_, g/kg DM	LOQ	LOQ
Ins(1,2,3,4)P_4_, g/kg DM	LOD	LOQ
Vicine, g/kg DM	0.63	0.02
Convicine, g/kg DM	0.03	-

1 LOQ, below limit of quantification (<0.17 g/kg DM for Ins(1,2,3,4,6)P_5_; <0.15 g/kg DM for Ins(1,2,5,6)P_4_; <0.10 g/kg DM for Ins(1,2,3,4)P_4_).

2 LOD, below limit of detection (<0.12 g/kg DM for Ins(1,3,4,5,6)P_5_; <0.06 g/kg DM for Ins(1,2,3,4,6)P_5_; <0.05 g/kg DM for Ins(1,2,3,4)P_4_). Other isomers were below the detection limit.

### Bird husbandry

All animal care procedures were approved by the institutional animal care and use committee of the Natural Resources Institute Finland before the beginning of the experiment. A total of 360 male Ross308® broiler chicks (DanHatch Ltd., Mynämäki, Finland) were utilized. Birds were housed in an environmentally controlled room, where conditions were set according to the breeder management guide (Aviagen, 2019). Throughout the entire pre-experimental and experimental period, birds were reared in pens (1 m length × 0.75 m height × 2 m width) covered by peat as litter and equipped with hanging feeders and nipple drinkers, which provided free access to feed and water. During the pre-experimental period, chicks were fed pelleted wheat-soybean based diets without exogenous enzymes supplementation, formulated to meet or exceed the Ross® Broiler Nutrition Specifications (Aviagen, 2019).

### Experimental design and diets

On d 21 post-hatch, a total of 360 birds (initial body weight, 1062 ± 35 g) were randomly assigned to six treatments with six replicates of ten birds each. The raw and dried crimped-ensiled faba bean seeds were included in the experimental diets as the main P source at the inclusion levels of 260, 403, and 546 g/kg (Table 2). The faba bean inclusion levels increased gradually, replacing corn starch, to achieve dietary P increments of 0.075 and 0.15 %, following recommendations of WPSA (2013). Corn starch was selected as one of the main ingredients in experimental feeds to ensure faba bean seeds served as the main dietary P source. To avoid potential interactions between Ca and P on bird responses, the total Ca:P ratio in all experimental diets was maintained at 1.4:1. According to the WPSA (2013) protocol, experimental diets should remain unchanged except for the inclusion of the P source under evaluation, i.e., raw and crimped-ensiled faba bean seeds. The method recommends that the ingredient under study must necessarily replace a phosphorus-free ingredient, in this study, corn starch. Given the differences in the nutritional composition of faba bean seeds and corn starch, changes in the concentrations of metabolizable energy (ME), and nutrients like crude protein (CP) and amino acids (AA) are expected and unavoidable as the inclusion levels of faba bean gradually increase in feeds.

**Table 2:** Ingredients and chemical composition of experimental faba bean diets, as-fed basis.

	RF^1^1	RF2	RF3	CEF^2^1	CEF2	CEF3
Ingredients, g/kg						
Corn starch	481.3	336.1	190.8	481.3	336.1	190.8
Albumin	180.0	180.0	180.0	180.0	180.0	180.0
Faba bean	260.0	403.0	546.0	260.0	403.0	546.0
Rapeseed oil	10.0	10.0	10.0	10.0	10.0	10.0
Limestone	6.2	8.4	10.7	6.2	8.4	10.7
Monocalcium phosphate	3.5	3.5	3.5	3.5	3.5	3.5
Salt (NaCl)	5.0	5.0	5.0	5.0	5.0	5.0
Choline chloride (60 %)	3.0	3.0	3.0	3.0	3.0	3.0
Dextrose	40.0	40.0	40.0	40.0	40.0	40.0
Vitamin supplement^3^	3.0	3.0	3.0	3.0	3.0	3.0
Minerals supplement^4^	3.0	3.0	3.0	3.0	3.0	3.0
Titanium dioxide	5.0	5.0	5.0	5.0	5.0	5.0
Calculated nutrient composition						
AME, kcal/kg	3270	3120	2970	3270	3120	2970
Crude protein, g/kg	220	257	293	225	264	302
Crude fat, g/kg	13.6	15.6	17.6	13.6	15.6	17.6
Calcium, g/kg	3.40	4.44	5.49	3.40	4.44	5.49
Available phosphorus^5^, g/kg	1.50	1.54	2.29	1.50	1.54	2.29
Total phosphorus, g/kg	2.42	3.17	3.92	2.42	3.17	3.92
Total indispensable amino acids calculated, g/kg						
Arginine	14.7	18.5	22.4	13.4	16.3	19.3
Histidine	5.2	6.2	7.2	5.4	6.5	7.6
Isoleucine	10.0	11.6	13.2	10.6	12.2	13.9
Leucine	17.5	20.5	23.5	18.2	21.4	24.5
Lysine	15.0	17.9	20.9	15.4	18.4	21.4
Methionine	5.5	5.8	6.1	5.8	6.1	6.4
Phenylalanine	11.5	13.2	14.9	12.0	13.8	15.6
Threonine	9.1	10.5	12.0	9.5	11.0	12.5
Tryptophan	0.9	1.3	1.6	0.9	1.3	1.7
Valine	13.1	15.0	16.9	13.6	15.6	17.5
Analyzed nutrient composition,						
Dry matter, g/kg	89.7	89.8	89.3	90.1	90.6	90.8
Gross energy, kcal/kg DM	4398	4426	4465	4357	4441	4491
Crude fiber, g/kg DM	33.8	42.4	57.5	30.6	42.7	65.1
Neutral detergent fiber, g/kg DM	50.5	67.6	90.2	41.2	53.8	77.2
Acid detergent fiber, g/kg DM	32.1	45.3	64.3	33.4	43.8	56.8
Total phosphorus, g/kg DM	2.37	3.14	4.09	2.64	3.39	4.41
InsP_6_-phosphorus, g/kg DM	0.71	1.06	1.41	0.71	1.02	1.43
InsP_6_, g/kg DM	2.51	3.76	5.02	2.51	3.63	5.08
Ins(1,2,4,5,6)P_5_, g/kg DM	0.23	0.35	0.46	0.17	0.23	0.35
Ins(1,2,3,4,5)P_5_, g/kg DM	LOQ^6^	LOQ	0.12	LOQ	0.12	0.17
Ins(1,2,5,6)P_4_, g/kg DM	LOD^7^	LOD	LOD	LOD	LOQ	LOQ

1 RF, raw faba bean.

2 CEF, crimped-ensiled faba bean.

3 Vitamin supplement supplied the following per kilogram of complete diet: vitamin A (acetate), 13,000 IU; vitamin D_3_, 5,000 IU; vitamin E (DL-α-tocopherol acetate), 80 IU; vitamin K_3_, 4 mg; vitamin B_1_, 4 mg; vitamin B_2_, 10 mg; vitamin B_6_, 6 mg; vitamin B_12_, 20 µg; calcium pantothenate, 20 mg; folic acid, 2 mg; biotin, 200 mg; niacin, 60 mg.

4 Mineral supplement supplied the following per kilogram of complete diet: Zn (zinc oxide), 100 mg; Mn (manganese sulfate), 120 mg; Fe (iron sulfate), 60 mg; Cu (copper sulfate), 16 mg; Co (cobalt carbonate), 1,000 µg; I (calcium iodate), 1.25 mg; Se (sodium selenite), 300 µg.

5 Available phosphorus: values calculated based on the analyzed total phosphorus content of the albumin (1.10 g/kg), corn starch (0.15 g/kg), monocalcium phosphate (214 g/kg) and faba bean (4.84 g/kg, considering 48 % of available phosphorus as described by Adekoya and Adeola (2023)).

6 LOQ, below limit of quantification (<0.12 g/kg DM for Ins(1,2,3,4,5)P_5_; <0.15 g/kg DM for Ins(1,2,5,6)P_4_).

7 LOD, below limit of detection (<0.10 g/kg DM for Ins(1,2,5,6)P_4_).

Titanium dioxide was included in the diets as an indigestible marker at 5 g/kg as-fed basis. The diets were provided in mash form and offered for *ad libitum* consumption throughout the 7-d assay (d 21 to 28 post-hatch). The raw and crimped-ensiled seeds were analyzed for DM, gross energy (GE), nitrogen (N), calcium (Ca), and P prior to diet formulation and manufacturing.

### Measurements, sample collection and chemical analysis

From d 25 to 28 post-hatch, rubber mats were placed on the floor of each pen to prevent litter consumption by the birds. Throughout this period, excreta produced by broilers were removed daily to maintain adequate hygienic conditions. On d 28 post-hatch, all birds and feed leftovers were weighed to determine body weight (BW), average daily feed intake (ADFI), average daily gain (ADG) and feed conversion ratio (FCR). At the end of the trial, all birds were sacrificed by cervical dislocation and immediately dissected, so that the ileum (section between Meckel´s diverticulum and 2 cm proximal to the ileocecal junction) could be excised. Digesta content from the distal half of the ileum was flushed out using distilled water into plastic containers. Digesta content from all the birds within the same pen was pooled and then stored at −80°C prior to lyophilization. Faba bean seeds and experimental diet samples were dried at 60°C, milled through a 1-mm mesh, and analyzed for DM (AOAC, 2006: method 934.01), P (AOAC, 2006: method 946.06) using inductively coupled plasma-optical emission spectroscopy (PerkinElmer Optima 8300 ICP-OES Spectrometer, PerkinElmer Inc., NC., Waltham, MA, USA), and titanium (Van Bussel et al., 2010) using the closed wet sulfuric acid and hydrogen peroxide digestion method in a microwave (CEM MDS 2000, CEM Corporation, Matthews, NC, USA), and the concentration was determined by inductively coupled plasma-optical emission spectroscopy (PerkinElmer Optima 8300 ICP-OES Spectrometer, PerkinElmer Inc., Waltham, MA, USA). Beans were also analyzed for GE using an adiabatic bomb calorimeter (C6010, IKA-Werke GmbH & Co. KG, Staufen, Germany) with benzoic acid as a standard, N by the combustion method (LECO Corporation, St Joseph, MI, USA; AOAC, 2019; method 986.06), and Ca (AOAC, 2006: method 985.01). Nitrogen content was multiplied by 6.25 to obtain crude protein. Lyophilized ileal digesta samples were analyzed for DM, P, and Ti content according to methodologies afore mentioned. Inositol phosphate isomers (InsP) in the beans, diets, and ileal digesta samples were determined by high-performance ion chromatography (ICS-3000 system; Dionex, Idstein, Germany) as described by Zeller et al. (2015) with minor modifications (Sommerfeld et al., 2018).

### Calculations and statistical analysis

The pre-cecal digestibility or disappearance of nutrients (DM, P, and InsP_6_) was calculated as follows:

$$pcdN\;\left(\%\right)=100-\left[100\;\times \frac{\left(marker_{diet}\times N_{dig}\right)}{\left(marker_{dig}\times N_{diet}\right)}\right]$$

where N_diet_ is the nutrient content in the diet (mg/kg DM), N_dig_ is the nutrient content in the digesta (mg/kg DM), *marker_diet_* is the titanium content in the diet (mg/kg DM), and *marker_dig_* is the titanium content in the digesta (mg/kg DM).

The amount of pcdN (g/kg of diet) was calculated by multiplying of the nutrient concentration in the diet (g/kg DM) and the respective pcdN (%) and dividing by 100.

The pre-cecal digestibility of phosphorus (pcdP) in diets (g/kg diet) was plotted against the P concentration increments (g/kg diet) in experimental diets according to WPSA (2013). A linear regression analysis was conducted, and the slope of the regression line was multiplied by 100 to determine the pcdP from the investigated source. The data were analyzed using analysis of variance (ANOVA) with the General Linear Model (GLM) procedure in SAS (Statistic Analysis System 9.4, SAS Institute Inc., Cary, NC, USA) considering diet, process (raw and crimped-ensiled), level of inclusion (L1, L2, and L3), and interaction of the process and inclusion level as fixed factors and replicate as random effect. The statistical model was:

y=μ+α+β+γ+βγ+rep+e

where µ= general mean; α= diet effect; β= processing method effect within diet; γ= inclusion level effect within diet; βγ= interaction between processing method and inclusion level; *rep*= replicate effect, and *e*= error of observation *y*. Replicates served as the experiment unit, and outliers were checked using the UNIVARIATE procedure of SAS. In all instances, differences were reported as significant at P-value equal to or less than 0.05. The linear effect of faba bean inclusion was tested using contrasts. Comparisons among treatment means were performed using Tukey’s multiple comparison test at a probability level of *P* < 0.05. Regression analysis was conducted using the GLM procedure, and the regression equation was generated with the “Solution I” option in SAS.

## Results

**Fig. 1:**
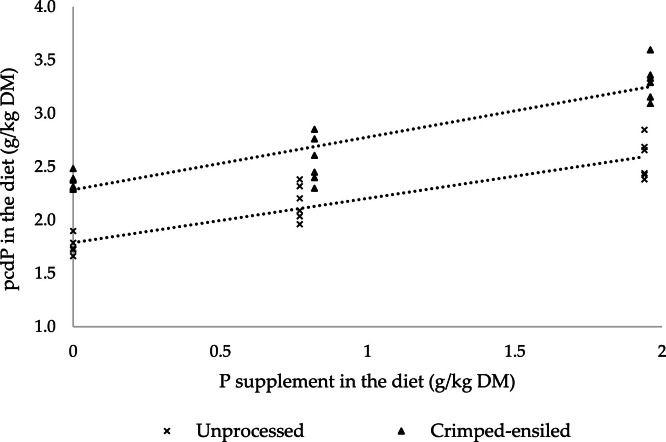
Linear regression analysis of pre-cecal digestibility P in diets (g/kg DM) plotted against dietary P increments from the raw and crimped-ensiled faba bean cv. Vire (g/kg DM). Values on the x-axis are based on analyzed dietary P concentration. Linear regression for raw and crimped-ensiled faba beans: *y* = 0.42x + 1.79 (R^2^ = 0.84; Sy.*x* = 0.15) and *y* = 0.49x + 2.28 (R^2^ = 0.83; Sy.*x* = 0.18).

The crimping-ensiling process decreased the concentration of V + C in faba bean cv. Vire to nearly zero (Table 1). As detailed in Table 2, the analyzed dietary P concentrations for the formulated levels of 2.42, 3.17, and 3.92 g P/kg were 2.37, 3.14, and 4.09 g P/kg, respectively, in the feeds containing raw seeds, and 2.64, 3.39, and 4.41 g P/kg in feeds with crimped-ensiled seeds. All results and regression data detailed herein were expressed based on analyzed values. No interactive effects between processing methods and faba bean inclusion level were observed on most of the assessed responses (*p* > 0.05; Table 3) except for the concentration of Ins(1,2,3,4,5)P_5_ and InsP_6_ in broiler ileal digesta (*p* < 0.01; Table 4). Irrespective of the processing method, increasing faba bean seed inclusion in feeds linearly decreased the pre-cecal disappearance of InsP_6_ in broiler chickens (*p* < 0.01; Table 3). Nonetheless, pre-cecal InsP_6_ disappearance in broilers fed crimped-ensiled faba bean seeds was higher (36.8 vs. 46.0 %) compared to birds fed raw seeds (*p* < 0.01). The concentration of InsP_4_ in broiler ileal digesta was below the limit of quantification (0.100 g/kg), except for Ins(1,2,3,4)P_4_, whose content in the digesta of birds fed the highest level (546 g/kg) of crimped-ensiled beans was 0.125 g/kg. No other inositol phosphates were detected in ileal digesta samples. The presence of inositol phosphates in the experimental feeds, but not in the distal ileal digesta, indicates that they were degraded along the gastrointestinal tract. Irrespective of the processing method, P intake increased with higher inclusions of faba bean in experimental feeds (*p* < 0.01; Table 3). Regardless of processing method, pcd of P decreased (*p* < 0.01) as faba bean inclusion increased from 260 to 403 g/kg (73.3 vs. 64.9 %), but no further reductions were detected between 403 and 546 g/kg (*p* > 0.05). Broilers fed crimped-ensiled seeds showed higher pcd of P (72.1 vs. 61.3 %) compared to those fed raw seeds (*p* < 0.01). As detailed in the Fig. 1, the pcd of P of the raw and crimped-ensiled faba bean seeds were estimated at 42 % and 49 %, respectively. Final BW, ADG, and FCR increased (*p* < 0.01) as dietary faba bean inclusion increased from 260 to 403 g/kg and stabilized (*p* > 0.05) between 403 and 546 g/kg inclusion levels. Overall, broilers fed crimped-ensiled faba bean seeds exhibited a higher ADG (59.9 vs. 55.3 g/d), a lower ADFI (90.5 vs. 96.7 g/d), and a better FCR (1.53 vs. 1.76) compared with birds fed diets containing raw seeds (*p* < 0.05). Orthogonal polynomial contrast analysis of broiler responses to increasing faba bean levels revealed linear improvement (*p* < 0.01) in final BW, ADG, and FCR for diets containing crimped-ensiled faba beans. In contrast, no significant effects (*p* > 0.05) on these performance traits were observed for raw faba bean inclusion levels.

**Table 3:** Performance and pre-cecal digestibility/disappearance (pcd) of total phosphorus and InsP_6_ in broilers fed diets containing raw or crimped-ensiled faba bean seeds.

Item	RF^1^^,^^4^			CEF^1^^,^^5^			L^2^^,^^6^			PR^3^^,^^7^		SEM	P-values			Linear (P-values)	
	L1	L2	L3	L1	L2	L3	1	2	3	RF	CEF		PR	L	PR*L	RF	CEF
Initial BW, g	1064	1059	1064	1062	1063	1064	1063	1061	1064	1062	1063	5.7	0.972	0.987	0.981	0.995	0.923
Final BW, g	1373	1410	1401	1369	1443	1468	1371^b^	1426^a^	1431^a^	1394	1425	9.9	0.07	0.01	0.278	0.374	<0.01
ADG, g/d	51.5	58.4	56.2	51.3	63.3	65.2	51.4^b^	60.8^a^	60.7^a^	55.3	59.9	1.3	0.042	<0.01	0.247	0.285	<0.01
ADFI, g/d	93.5	97.8	98.7	86	92.8	93.2	89.8	95.3	95.9	96.7	90.5	1.3	0.016	0.072	0.868	0.281	0.071
FCR, g/g^8^	1.84	1.68	1.76	1.68	1.47	1.44	1.76^b^	1.58^a^	1.60^a^	1.76	1.53	0.03	<0.01	<0.01	0.203	0.398	<0.01
P intake, mg/d^9^	222	307	403	226	315	376	224^c^	311^b^	389^a^	311	306	13.3	0.732	<0.01	0.543	<0.01	<0.01
pcd P, %	66.2	61.6	56.1	80.5	68.2	67.5	73.3^a^	64.9^b^	61.8^b^	61.3	72.1	1.41	<0.01	<0.01	0.084	<0.01	<0.01
pcd InsP_6_ disappearance, %	42.9	41.8	25.8	57.5	42.5	29.4	50.2^a^	43.2^a^	27.2^b^	36.8^b^	46.0^a^	2.47	0.047	<0.01	0.307	0.021	<0.01

1 Each value represents the mean of six replicate pens of 10 birds each.

2 Each value represents the mean of twelve replicate pens of 10 birds each.

3 Each value represents the mean of eighteen replicate pens of 10 birds each.

4 RF, raw faba bean.

5 CEF, crimped-ensiled faba bean.

6 L, inclusion level.

7 PR, processing method.

8 FCR, feed conversion ratio.

9 P intake, total phosphorus intake.

a-c Values with different superscript letter in a row were significantly different at 5 % Tukey test.

**Table 4:** Concentration of InsP isomers in the ileal digesta of broilers fed diets containing raw or crimped-ensiled faba bean seeds.

		Ins(…)P_5,_ g/kg DM			InsP_6,_ g/kg DM
Faba beans	Level	(1,2,3,4,6)^1^	(1,2,3,4,5)	(1,2,4,5,6)	
Raw	1	LOQ^2^	0.26^c^	0.16^c^	6.86^c^^d^
	2	LOQ	0.34^b^^c^	0.24^b^^c^	8.32^b^^c^^d^
	3	LOQ	0.42^b^	0.33^a^^b^	10.6^b^
Crimped-ensiled	1	LOQ	0.24^c^	0.16^c^	6.27^d^
	2	LOQ	0.39^b^	0.25^b^^c^	9.31^b^^c^
	3	0.27	0.64^a^	0.39^a^	14.8^a^
Main effects					
Raw		-	0.34	0.24	8.58
Crimped-ensiled		-	0.40	0.32	9.83
Level 1		-	0.25^c^	0.16^b^	6.56^c^
Level 2		-	0.37^b^	0.24^b^	8.78^b^
Level 3		-	0.50^a^	0.36^a^	12.5^a^
SEM			0.04	0.03	0.78
*P-values*					
Process		-	<0.01	0.255	<0.01
Level		-	<0.01	<0.01	<0.01
Process × Level		-	<0.01	0.391	<0.01

1 No P-values given due to values under the limit of quantification.

2 LOQ, below limit of quantification (<0.17 g/kg DM for Ins(1,2,3,4,6)P_5_).

a-d Values with different superscript letter in a column were significantly different at 5 % Tukey test.

## Discussion

Expectedly, the concentration of V + C in faba bean cv. Vire was low and decreased to nearly zero in crimped-ensiled seeds. Similar results were reported by Rinne et al. (2020), who found that crimping and ensiling were effective to degrade completely vicine in faba bean and decrease by 90 % the convicine concentration in seeds. The current study investigated the effects of crimping and ensiling on the dephosphorylation of InsP_6_ in faba bean seeds, the pre-cecal InsP_6_ disappearance, and the digestibility of P in broilers. The pcd of P was evaluated using the protocol described by WPSA (2013). We hypothesized that InsP_6_ present in faba bean seeds would be hydrolyzed to some extent into lower InsP isomers by the crimping and ensiling process, which could consequently improve the digestibility of P in seeds. Overall, our hypothesis was confirmed by the results, although the magnitude of the crimping and ensiling process was smaller than initially expected. Crimping and ensiling reduced the InsP_6_ concentration of the beans from 2.6 to 2.4 g InsP_6_-P/kg DM and increased the pre-cecal InsP_6_ disappearance for the diets from 37 % to 46 % and pcd of P in the beans from 42 % to 49 %.

The relatively small InsP₆ degradation observed in the crimped-ensiled beans may be attributed to the activation of plant intrinsic phytases during moistening prior to crimping and ensiling. It has been shown that soaking initiates physiological changes in seeds, notably the activation of enzymes, such as phytases, which prepare the seed for germination (Bau et al., 1997). Literature indicates, however, that although soaking can reactivate plant enzymes, the activity of phytate-degrading enzymes in faba bean and other grain legumes becomes substantially higher during the actual germination stage (Egli et al., 2002; Luo et al., 2012). The intrinsic phytate-degrading enzyme of faba bean has been characterized as a monomeric protein with a single pH optimum at 5.0 and an optimal temperature of 50 °C (Greiner et al., 2001). In a subsequent publication, the aforementioned research group demonstrated that faba bean endogenous phytase degrades phytate via D-Ins(1,2,3,5,6)P_5_, D-Ins(1,2,5,6)P_4_, D-Ins(1,2,6)P_3_, and D-Ins(1,2)P_2_ to finally Ins(2)P, and also exhibits low 3-phytase activity (Greiner et al., 2002). Although the analytical method used in the current study differs from that of the referred authors, the InsP isomers detected herein are consistent with those previously reported. As shown in Table 2, we noticed that feeds containing the intermediate and the highest inclusion of crimped–ensiled beans exhibited higher concentrations of Ins(1,2,3,4,5)P_5_ and Ins(1,2,5,6)P_4_ compared to those with raw beans, supporting the hypothesis that endogenous phytase activity was, to some extent, induced during processing. Nonetheless, as the beans investigated in the current study were ensiled prior to germination, InsP_6_ hydrolysis by endogenous plant enzymes was presumably limited. Microbial phytases from lactic-acid-producing bacteria might have influenced InsP_6_ degradation in crimped-ensiled beans.

The increase in InsP_6_ content of the diets by increased bean inclusion markedly decreased the percentage of InsP_6_ disappearance for the diets. Such findings are consistent with other studies that varied the InsP_6_ content of diets not supplemented with exogenous phytases (Rodehutscord et al., 2017; Dersjant-Li et al., 2019; Haetinger and Adeola, 2024; Q. Zhang et al., 2024). InsP_6_ hydrolysis is catalyzed by endogenous phosphatases at such conditions, but their activities are limited. In the present study, for the three different inclusion levels of crimped-ensiled beans they degraded a similar amount of InsP_6_ in feeds (1.44; 1.54; and 1.49 g/kg DM), indicating that the endogenous hydrolysis capacity had been reached already at the lowest bean inclusion level. Interestingly, in feeds containing raw beans, the amount of degraded InsP_6_ in the lowest level under study increased from 1.08 to 1.57 g/kg DM but decreased to 1.29 g/kg DM in the highest bean level.

Although not characterized in this study, the microbial community in the crimped–ensiled beans, as in other ensiled materials, naturally included a diverse microbial community capable of expressing fibrolytic enzymes. These enzymes may have contributed to the hydrolysis of cell wall structures, increasing the accessibility of previously encapsulated fractions of InsP₆ and other nutrients to intestinal degradation. This enhanced substrate accessibility, and the potential synergy between intrinsic phytate-degrading enzymes in faba beans and those of microbial origin, may explain the higher InsP₆ hydrolysis observed in birds fed the lowest inclusion level of crimped–ensiled beans. In contrast, birds fed raw beans likely relied mainly on intestinal endogenous phytases and phosphatases to hydrolyze InsP₆, much of which was encapsulated within cell walls. The lower hydrolysis capacity and restricted substrate availability may account for the gradual increase in the hydrolysis capacity of InsP₆ observed from the lowest to the intermediate inclusion level. At the highest inclusion level, where faba beans comprised more than half of the diet, we noticed that dietary InsP₆ hydrolysis declined. This reduction could be attributed to the elevated concentration of ANFs, particularly non-starch polysaccharides, which have been extensively shown to increase digesta viscosity, compromising nutrient digestibility and absorption (Bedford et al., 2024).

The linear increment observed on P intake in birds fed increasing levels of raw and crimped-ensiled faba beans was expected as the concentration of P in experimental diets increased but ADFI remained unaffected by the inclusion of seeds in feeds. Irrespective of the processing method, linear reductions in the pcd of P were observed with increasing levels of faba bean in feeds. The decrease in the pcd of P might be related to the nature of the P present in the ingredients used in feed formulation. Except for the faba bean seeds that contained approximately half of their total P in the form of InsP_6_, the main feed ingredients utilized in the experimental feeds contained as little P as possible [albumin (1.26 mg P/kg DM), corn starch (0.18g/kg DM) and dextrose (undetected P)], and negligible amounts of phytate P. As outlined in Table 2, the dietary content of phytate P nearly doubled as the inclusion of raw and crimped-ensiled beans in feeds increased from 260 to 546 g/kg. The lower digestibility of P in faba bean seeds, especially phytate P, compared to such feed ingredients and monocalcium phosphate may explain the impairments caused by higher inclusion levels. Our findings support previous research that reported linear reductions in pcd of P in broilers fed increasing dietary soybean meal inclusions (Rodehutscord et al., 2017; Haetinger and Adeola, 2024; Haetinger et al., 2024). Conversely, the outcomes found herein differ from those reported by Adekoya & Adeola (2023), who did not note any effect of increasing faba bean inclusions on the apparent digestibility of P in broiler chickens.

We determined the pcd of P of the raw and crimped-ensiled faba bean cv. Vire as 42 and 49 %, respectively (Fig. 1). As detailed in Table 2, the experimental feeds whose concentration of P were calculated as 2.42, 3.17, and 3.92 g P/kg contained 2.37, 3.14, and 4.09 g P/kg, respectively, in the feeds containing raw seeds, and 2.64, 3.39, and 4.41 g P/kg in crimped-ensiled seeds. All results and regression data were expressed based on analyzed P basis. This is the first trial whereby the pcd of P of faba bean in broilers is determined according to the WPSA (2013) protocol and the impacts of fermentation on pcd P digestibility is investigated. Therefore, our findings could hardly be compared with existing literature on faba beans. To the best of our knowledge, the only study that addressed the ileal digestibility of P in faba beans was conducted by Adekoya and Adeola (2023), who reported a true ileal digestibility value of 67 % in broilers. The difference between the reference and our coefficient is considerably large. Variations in the inclusion levels of monocalcium phosphate and limestone, as well as differences in the dietary concentrations of calcium and P, may help explain this discrepancy. While the pcd of P observed herein for faba bean is lower than that reported by the aforementioned authors, it is comparable to values reported for ingredients like soybean meal (42 %−46 %;Mirabile et al., 2020; Haetinger and Adeola, 2023; Haetinger et al., 2024) and higher than that determined for black-eyed peas (29 %; Iyayi et al., 2013). Olowe and Adeola (2025) has recently reported the apparent ileal and apparent total tract digestibility of phosphorus in raw faba bean seeds fed to broilers as 52 % and 54 %, respectively.

Although the concentration of ME and CP in experimental feeds changed as raw and crimped-ensiled faba bean increased, ADFI was not affected by the levels under study. Broilers fed crimped-ensiled seeds had lower ADFI compared to those fed raw seeds, which may be explained by the higher gross energy content of crimped-ensiled seeds. Despite higher ADFI, broilers fed raw seeds exhibited lower ADG than those fed crimped-ensiled faba bean seeds, which showed linear improvements in ADG as faba bean inclusion increased. The improved growth responses in birds fed crimped-ensiled seeds may be attributed to the reduction of ANFs, which likely enhanced the pcd of DM and P. Additionally, the increased dietary P concentration in crimped-ensiled feeds may have contributed to these improvements. However, this effect was not observed in broilers fed raw seeds, possibly due to the higher concentration of ANFs in their diets. Although FCR improved linearly with increasing faba bean inclusion regardless of the processing method, broilers fed raw faba beans exhibited poorer FCR compared to those fed crimped-ensiled seeds. This outcome is consistent with the greater increase in ADFI relative to ADG observed in the group of birds fed raw seeds.

## Conclusions

Crimping and ensiling did not substantially affect extent the InsP_6_ content of faba beans but increased the pre-cecal disappearance of InsP_6_ and the pre-cecal P digestibility of the seeds compared to raw seeds when fed to broilers.

## CRediT authorship contribution statement

**Letícia Soares:** Writing – review & editing, Writing – original draft, Visualization, Validation, Supervision, Resources, Project administration, Methodology, Investigation, Funding acquisition, Formal analysis, Data curation, Conceptualization. **Heidi Högel:** Writing – review & editing, Investigation, Funding acquisition, Conceptualization. **Liisa Keto:** Writing – review & editing, Resources, Conceptualization. **Marcia Franco:** Writing – review & editing, Conceptualization. **Marketta Rinne:** Writing – review & editing, Writing – original draft, Methodology, Investigation, Funding acquisition, Conceptualization. **Markus Rodehutscord:** Writing – review & editing, Writing – original draft, Resources, Methodology, Data curation. **Gabriel da Silva Viana:** Writing – review & editing, Writing – original draft, Visualization, Validation, Supervision, Resources, Project administration, Methodology, Investigation, Funding acquisition, Formal analysis, Data curation, Conceptualization.

## Disclosures

The authors declare that they have no known competing financial interests or personal relationships that could have appeared to influence the work reported in this paper.
